# From Monoamine Oxidase Inhibition to Antiproliferative Activity: New Biological Perspectives for Polyamine Analogs

**DOI:** 10.3390/molecules28176329

**Published:** 2023-08-29

**Authors:** Giulia Nordio, Francesco Piazzola, Giorgio Cozza, Monica Rossetto, Manuela Cervelli, Anna Minarini, Filippo Basagni, Elisa Tassinari, Lisa Dalla Via, Andrea Milelli, Maria Luisa Di Paolo

**Affiliations:** 1 Department of Pharmaceutical and Pharmacological Sciences, University of Padova, 35131 Padova, Italy; giulia.nordio.1@phd.unipd.it (G.N.); francesco.piazzola@studenti.unipd.it (F.P.); lisa.dallavia@unipd.it (L.D.V.); 2 Consorzio Interuniversitario Nazionale per la Scienza e Tecnologia dei Materiali (INSTM), 50121 Firenze, Italy; 3 Department of Molecular Medicine, University of Padova, 35131 Padova, Italy; giorgio.cozza@unipd.it (G.C.); monica.rossetto@unipd.it (M.R.); 4 Department of Science, University of Rome “Roma Tre”, 00146 Rome, Italy; manuela.cervelli@uniroma3.it; 5 Department of Pharmacy and Biotechnology, Alma Mater Studiorum-University of Bologna, 40126 Bologna, Italy; anna.minarini@unibo.it (A.M.); filippo.basagni2@unibo.it (F.B.); 6 Department for Life Quality Studies, Alma Mater Studiorum-University of Bologna, 47921 Rimini, Italy; elisa.tassinari9@unibo.it

**Keywords:** polyamine analogs, monoamine oxidases, antiproliferative activity, inhibitors, docking studies, LN-229 cells

## Abstract

Monoamine oxidases (MAOs) are well-known pharmacological targets in neurological and neurodegenerative diseases. However, recent studies have revealed a new role for MAOs in certain types of cancer such as glioblastoma and prostate cancer, in which they have been found overexpressed. This finding is opening new frontiers for MAO inhibitors as potential antiproliferative agents. In light of our previous studies demonstrating how a polyamine scaffold can act as MAO inhibitor, our aim was to search for novel analogs with greater inhibitory potency for human MAOs and possibly with antiproliferative activity. A small in-house library of polyamine analogs (**2**–**7**) was selected to investigate the effect of constrained linkers between the inner amine functions of a polyamine backbone on the inhibitory potency. Compounds **4** and **5**, characterized by a dianiline (**4**) or dianilide (**5**) moiety, emerged as the most potent, reversible, and mainly competitive MAO inhibitors (Ki < 1 μM). Additionally, they exhibited a high antiproliferative activity in the LN-229 human glioblastoma cell line (GI_50_ < 1 μM). The scaffold of compound **5** could represent a potential starting point for future development of anticancer agents endowed with MAO inhibitory activity.

## 1. Introduction

Monoamine oxidases (MAOs) are mitochondrial-membrane-bound, FAD-dependent enzymes that catalyze the oxidative deamination of biogenic amines, in particular monoamine neurotransmitters such as serotonin, dopamine, and norepinephrine [1]. The two enzyme isoforms, monoamine oxidase A (MAO A) and monoamine oxidase B (MAO B), are characterized by different substrate and inhibitor specificities. MAOs are more common inside neurons and astroglial cells as part of the central nervous system (CNS), but they are also present in other organs. MAO A is preferentially expressed in the gastrointestinal tract, lung, liver, and placenta, while MAO B is found in the platelets [2].

Since they are involved in the catabolism of neurotransmitters, MAOs are well-known pharmacological targets in brain disorders. Several MAO inhibitors (MAOIs) have attained clinical approval for the treatment of major depressive disorder and Parkinson’s disease, as they promote an increase in the level of monoamine neurotransmitters [3,4]. Moreover, the therapeutic potential of MAOIs appears more valuable because an overactivity of MAOs can lead to an increased production of harmful species such as aldehydes and hydrogen peroxide [2]. Thus, the overexpression of MAO and the upregulation of its activity, which have been found in age-associated diseases, were suggested to contribute to the development of oxidative and inflammatory stress. This condition has been recently linked to the pathogenesis and progression of various diseases, from neurodegenerative to cardiovascular disorders to cancer [5].

MAOs have been recently found highly expressed in different types of cancer [6,7,8,9,10,11,12,13]. In particular, various studies have demonstrated a correlation between MAO A expression and the percentage of high-grade prostate cancer [11,14,15]. Patients with recurring castrate-sensitive prostate cancer have shown a significant measurable decline in the prostate-specific antigen after treatment with the irreversible non-selective MAO A/B inhibitor phenelzine [16]. Additionally, overexpression of MAO A was found in glioblastoma [17], and MAO A has also been suggested to contribute to the progression of non-small-cell lung cancer (NSCLC) due to its role in regulating the epithelial-to-mesenchymal transition (EMT) process, a key step in cancer invasion and metastasis [10,18]. Similarly, MAO B was found overexpressed in glioblastoma [12,19] and in colorectal cancer [20]. Therefore, this novel role for “old enzymes” is opening new frontiers for MAOIs as potential antiproliferative agents [21,22,23,24].

In past years, we focused our studies on polyamine derivatives as a novel type of MAOIs. Indeed, the polyamine skeleton represents a “universal template” since the insertion of appropriate moieties on amine groups and the type of linker between them can modulate selectivity and affinity toward a given receptor or enzyme [25,26]. This approach allowed the design and development of some polyamine analogs able to hit targets involved in cancer growth (from DNA to enzymes involved in the polyamine metabolism to receptors [27,28,29,30,31,32,33]) and other types of analogs able to hit targets involved in multi-factorial neurodegenerative diseases such as Alzheimer’s disease [25,34]. In this research area, we have highlighted that some polyamine-based analogs can inhibit amine oxidases, such as the human MAOs [35]. In particular, we have demonstrated that a decrease in the flexibility of the inner polymethylene chain of methoctramine (compound **1**), as in its dipiperidine analog **2**, is favorable to MAO B inhibitory activity. On this basis, by searching for novel compounds endowed with both MAO inhibitory potency and antiproliferative activity, we selected a small in-house library of polyamine analogs (**3**–**7**) structurally related to compound **2**. These analogs are characterized by constrained linkers between the inner amine functions of the polyamine backbone. The role of these substitutions on the MAO inhibitory activity and the antiproliferative potential of compounds **2**–**7** were investigated. Two of these analogs (**4** and **5**) were found to join these activities, demonstrating remarkable antiproliferative activity in LN-229, a human glioblastoma cell line.

## 2. Results

### 2.1. Rational of Compound Selection

In our previous studies, we reported a series of synthetic polyamines as new MAOIs [35]. In particular, the dipiperidine analog of compound **1**, the polyamine **2**, previously designed to act as a muscarinic cholinergic M2 receptor antagonist (compound **2**) [34] emerged as active and reversible mixed-type MAO B inhibitor.

**Figure 1:**
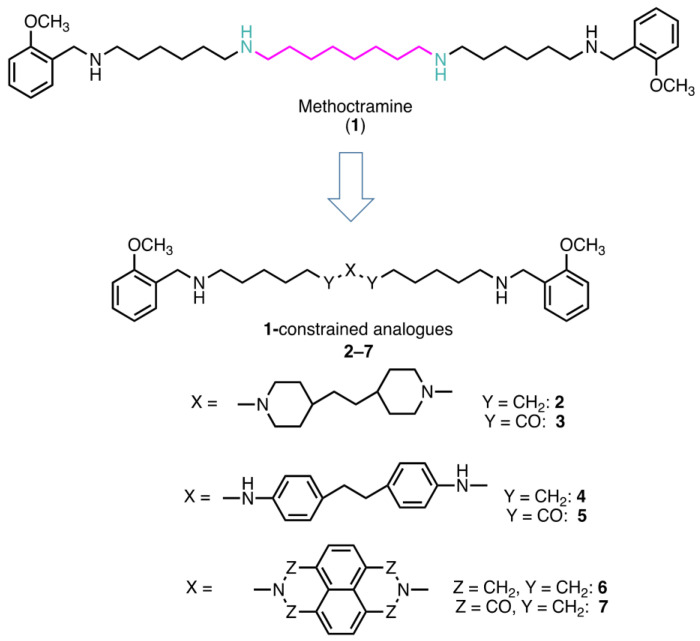
Methoctramine (**1**) and its constrained analogs **2**–**7**. Octamethylene linker replaced with constrained cycles is highlighted in pink. Inner basic nitrogens are highlighted in green.

Based on this and given our interest in discovering new MAOIs, in this study we evaluated compound **1** and the structurally related in-house constrained analogs **2**–**7** as human MAO inhibitors. From a chemical point of view, compounds **2**–**7** are characterized by conformationally restricted moieties between the inner nitrogen atoms of the polyamine skeleton with respect to the flexible inner eight-carbon chain of compound **1** (Figure 1). In detail, the flexible linker has been replaced with a dipiperidine (compounds **2** [34] and **3** [34]) or dianiline (compounds **4** [34] and **5** [34]) moiety or naphthalene diimide-related rings (compounds **6** and **7** [36]). It is worth noting that compounds **2**, **4**, and **6** are characterized by basic inner nitrogens, while compounds **3**, **5**, and **7** (bearing the corresponding amide or imide functional groups) are characterized by non-basic inner nitrogens.

### 2.2. Effects of the Polyamine Derivatives **2**–**7** on the Activity of Amine Oxidases

#### 2.2.1. Inhibitory Activity of the Compounds on Human Recombinant MAOs

Before evaluating the potential MAO inhibitory capability of derivatives **2**–**7**, kinetic experiments were performed to assay their behaviors as potential MAO substrates by using the Amplex Red/horseradish peroxidase coupled method, which detects the hydrogen peroxide produced by the amine oxidase enzyme activity in the presence of an amine substrate. Compounds **2**, **3**, and **5**–**7** (up to a 50 μM concentration) did not act as substrates, while **4** was not tested because of interferences with the assay procedure. Thus, for the subsequent inhibition studies, an assay method that used kynuramine as the MAO substrate and detected the production of 4-hydroxyquinoline (formed from the aldehyde reaction product) was applied.

**Figure 2:**
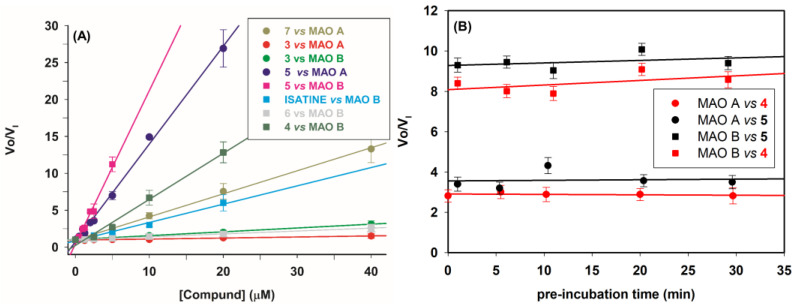
Effects of polyamine analogs **3**–**7** on MAO A and MAO B activity. (**A**) Effect of different concentrations (1.25–40 μM) of tested compounds on human recombinant MAO A (◯, circle) or MAO B (□, square) activity. The ratio between the velocity in the absence (V_o_) and in the presence (V_I_) of the tested compound (V_o_/V_I_) increased linearly with the concentration (r > 0.99) (**B**) Effect of incubation time with compounds **4** and **5** on MAO A and B activity. The (V_o_/V_I_) ratio was determined after different pre-incubation times (0–30 min) of the enzyme with the inhibitor (at 2.5 μM concentration) before the addition of the substrate (kynuramine, 10 μM).

The effect of derivatives **2**–**7** on MAO activity was assessed using human recombinant enzymes and compound concentrations in the range of 1.25–40 μM. Isatine, harmine, and safinamide were evaluated as known competitive MAOIs, while compound **1** was taken as the reference molecule. [37,38,39]. For this initial kinetic screening, the substrate (kynuramine) was used at a concentration (10 μM) lower than the K_M_ value (K_M_ = 32 ± 11 μM for MAO A and K_M_ = 27 ± 4 μM for MAO B). This experimental condition was chosen because, according to our earlier study [35], compound **2** mostly acted as a competitive inhibitor, thus affecting mainly K_M_ rather than V_max_. Consequently, under the selected experimental conditions of [S] = 10 μM < K_M_, the ratio of the initial rate of reaction in the absence (V_o_) and in the presence (V_I_) of the tested compound (V_o_/V_I_) could be roughly simplified to the ratio of (V_max_/K_M_)_o_/(V_max_/K_M_)_I_ ≈ (1 + [I]/Ki). In plotting the ratio (V_o_/V_I_) against the compound concentration, a linear dependence of V_o_/V_I_ on the inhibitor concentration should be obtained, and the Ki values could be calculated. This behavior was confirmed (see examples in Figure 2A), as the ratio (V_o_/V_I_) increased linearly with the concentration of all tested compounds (r > 0.99). Based on the slope values (1/Ki) calculated via the linear regression analysis, the inhibition constant (Ki) values of the tested compounds for both MAO A and MAO B were calculated along with the selectivity index SI; that is, the ratio Ki_MAO A_:Ki_MAO B_ (Table 1).

As shown in Table 1, the insertion of a less flexible chain into the compound **1** scaffold led to the appearance of an inhibitory effect on both isoenzymes that seemed strongly dependent on the nature of the linker. Specifically, for **2**, which was characterized by a dipiperidine moiety instead of the flexible eight-carbon chain between the inner nitrogen atoms, only a slight increase in MAO B inhibition was observed, while no appreciable effect was obtained toward MAO A (in accordance with our previous data [35]). The replacement of the inner dipiperidine moiety of **2** with a dianiline (compound **4**) strongly improved the inhibitory ability of the polyamine scaffold against both the MAO isoforms. Indeed, the Ki value calculated for MAO B decreased by more than two orders of magnitude, with Ki = 0.3 μM and 330 μM for **4** vs. **2**, respectively. Similarly, compound **4** also exerted a remarkable inhibition of MAO A (Ki = 0.9 μM), in contrast with **2**, which was basically inactive (Ki = 247 μM). The modification of the inner amine groups in amide functions had a further slight positive effect in ameliorating the inhibitory potency as assessed by the reduction in Ki values of compound **3** vs. compound **2** and compound **5** vs. compound **4**.

In addition, the replacement of the inner dipiperidine moiety (**2**) with a more rigid and bulky naphtalentetracarboxylic diimide core (**7**) resulted in a derivative that maintained the ability to inhibit both the MAO isoforms, even if with less potency than **5** (compound **7**, Ki = 3–4 μM; compound **5**, Ki < 1 μM). Finally, it is important to point out that modifying the basic inner nitrogen atoms of compound **6** in imide functions, as in compound **7**, an increase in the inhibitory capability was obtained (Ki decreased by about 5 (MAO B) to 10 (MAO A) times).

#### 2.2.2. Mechanism of Inhibition

To understand the mechanism of inhibition, additional investigations were focused on the most potent inhibitors (**4** and **5**). First, we evaluated the possibility of a time-dependence inhibition. For this purpose, the residual MAO activity was determined after pre-incubation at different time points (0 to 30 min) for both the human recombinant MAO A and MAO B with a 2.5 μM concentration of compounds **4** and **5** before adding kynuramine (10 μM) as the substrate. For both compounds, the Vo/V_I_ ratio did not change in the explored time range; that is, no time dependence of the inhibition was observed (Figure 2B).

Then, the reversibility of the inhibition was verified. In detail, MAO A and MAO B were incubated with compound **4** or **5** (5 μM) for 25 min, then the enzyme–inhibitor complexes were diluted 50 times, and the enzyme activity was assayed under a substrate-saturation condition (300 μM of kynuramine). The recovered enzyme activity was not significantly different in comparison to the control sample (in the absence of the inhibitor), confirming the full reversibility of the inhibition of MAO activity by this type of polyamine analog.

To further clarify the mechanism of inhibition, the kinetic parameters K_M_ and V_max_ of the human recombinant MAO A and MAO B were determined in the presence of various concentrations of compounds **4** or **5** (0.25–2 μM). Kynuramine was used as the substrate. The global fit analysis (Software GraphPad Prism 9.0) of the experimental data (velocity vs. substrate concentration at the various inhibitor concentrations) was applied by testing the equations for competitive, mixed, non-competitive, and uncompetitive inhibition models. The mixed model fit resulted in the highest r^2^ value, with a dissociation constant value for the enzyme–substrate complex (K_IES_) at least 17 times higher in comparison to the inhibition constants for the free enzyme (K_I_). Therefore, these compounds interacted preferentially with the active site of the free enzyme (MAO A or MAO B) in competition with the substrate. In detail, the following inhibition constant values were obtained: for **5**, K_I_ = 0.16 ± 0.04 μM and K_IES_ = 6 ± 1 μM vs. MAO B (r^2^ > 0.96) and K_I_ = 0.62 ± 0.09 μM and K_IES_ = 14 ± 2 μM vs. MAO A (r^2^ > 0.99); and for **4**, K_I_ = 0.31 ± 0.05 μM and K_IES_ = 15 ± 2 μM vs. MAO B (r^2^ > 0.97) and K_I_ = 0.62 ± 0.11 μM and K_IES_ = 11 ± 2 μM vs. MAO A (r^2^ > 0.99). These K_I_ values are in good agreement with those reported in Table 1.

**Figure 3:**
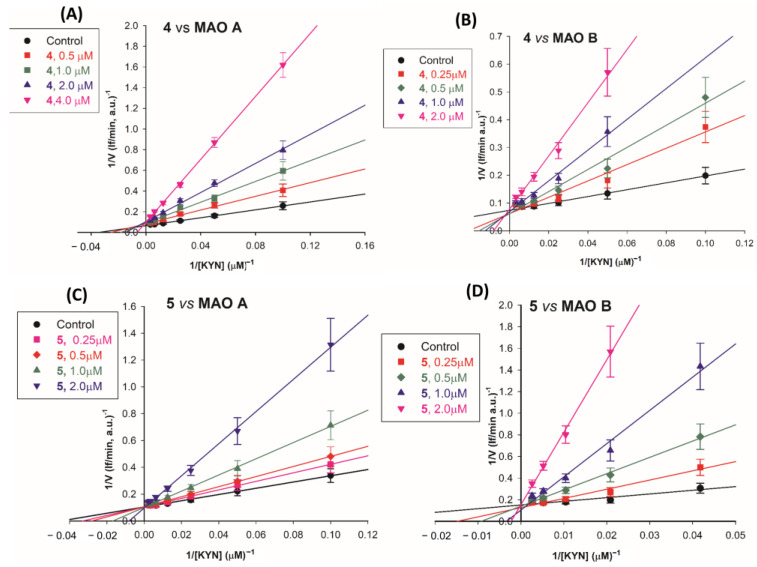
Mainly competitive inhibition of MAO A and MAO B by compounds **4** and **5**. Double reciprocal plots of MAO A (panels **A**,**C**) and of MAO B (panels **B**,**D**) activity in the absence (●, control) and in the presence of **4** or **5** (0.25–2 μM). Continuous lines are the results of the linear regression analysis of the plotted data (r > 0.99).

This primary competitive inhibition exerted by compounds **4** and **5** can be better visualized by the double reciprocal or Lineweaver–Burk plots (1/V vs. 1/S) of the kinetic data for MAO A and MAO B in the presence of the various concentrations of the inhibitors (Figure 3A–D). These plots clearly demonstrate that the compounds mostly affected the intercept on the *x*-axis (−1/K_M_), while the intercept on the *y*-axis (1/V_max_) was just marginally increased at the highest concentrations of 4 and **5**. The mainly competitive/mixed mode of action of these compounds agreed with that previously found for **2** and MAO B [35]. This mechanism of inhibition also was verified for compound **7**, as K_M_ was found to be more affected than the V_max_ parameter by the presence of the compound (Figure S1).

#### 2.2.3. Selectivity of Compounds **1**–**3** and **5**–**7** versus Other Amine Oxidases

In order to evaluate the potential effect of polyamine analogs as MAO inhibitors in cells, it is important to have information on their selectivity toward other types of amine oxidases (AOs). In our previous studies [35,40], compound **2** was found to be a poor inhibitor of the human Semicarbazide-Sensitive Amine Oxidase/Vascular Adhesion Protein-1 (VAP-1) and of murine spermine oxidase (SMOX), and compound **1** was found to be more effective than **2** for SMOX but very poorly active for VAP-1. These two AOs are important pharmacological targets: VAP-1 is involved in various inflammatory diseases and some types of cancer [41,42], and SMOX is involved in both the catabolism of polyamines (spermine and spermidine) and in the progression of some types of cancer [43,44]. Because of this, the specificities of compounds **3** and **5**–**7** toward these two other types of amine oxidases were evaluated. As reported above, due to the interferences with the amine oxidase assay method that detected the generation rate of hydrogen peroxide, it was not possible to test compound **4**, Thus, after verifying that the analogs **3** and **5**–**7** are not substrates of VAP-1 and SMOX (up to a 50 μM concentration), the compounds were tested at a fixed concentration as potential inhibitors. Their effects on the catalytic efficiency (V_max_/K_M_) are shown in Table 2, in which the V_max_/K_M_ relative to each control sample (V_max_/K_M_)_+i_/(V_max_/K_M_)_0_; that is, the residual catalytic efficiency in the presence of a specific concentration of the compound, are reported. This kinetic parameter provides information about the physical interaction of the compound with the enzyme under a non-saturating condition of the substrate. The results in Table 2 suggest that VAP-1 was poorly inhibited by all the tested compounds (at a 50 μM concentration): compound **3** appeared to be the most effective of the series, while for compound **5** (the most effective on MAO B), about 0.5 of the residual activity was found (a Ki of about 50 μM could be roughly calculated). In the case of SMOX, it was confirmed that compound **1** inhibited the enzyme with a Ki of about 10 μM [40], and compound **7**, which appeared to be the most effective of the series, was only slightly more effective than compound **1** in inhibiting the enzyme. It can be deduced that the substitution of the dipiperidine moiety with the dianiline–amide moiety (compound **5**) was very effective in increasing the inhibitory potency toward the two MAO isoforms by more than two orders of magnitude but was almost ineffective for VAP-1 and SMOX. Thus, compound **5** (and possibly, also compound **4**) can be considered as a potent and selective inhibitor of human MAOs.

### 2.3. Docking Studies

**Figure 4:**
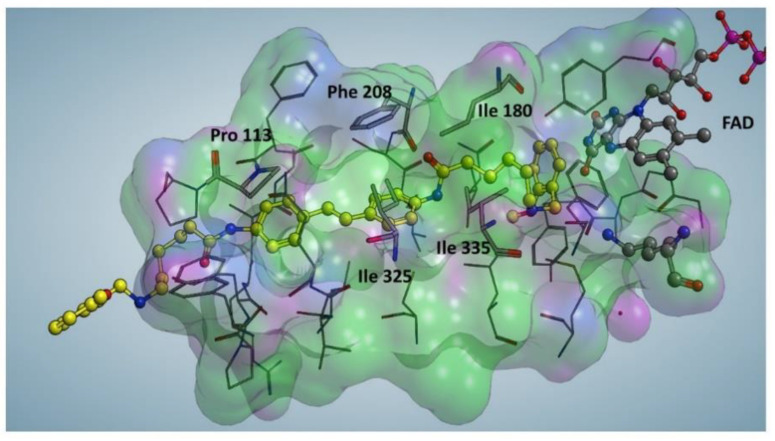
Docking simulation of compound **5** inside the catalytic site of MAO A. The catalytic pocket of MAO A is configured as a predominantly hydrophobic tunnel (green regions), while the polar portions (purple) mainly face the solvent. The molecule (yellow) is almost totally immersed inside the catalytic pocket of MAO A.

Compound **5**, the most potent MAO A and MAO B inhibitor, was docked against the MAO A crystal structure (PDB code: 2Z5Y) to determine its binding mode. Before the docking procedure, a Site Finder approach was applied to select all the possible binding sites of MAO A. Interestingly, the most suitable interaction region was superimposed on the catalytic binding site of MAO A, in accordance with the competitive mechanism of action of this molecule reported in the enzyme activity assays. The catalytic site of MAO A (as well as MAO B) is characterized by a hydrophobic tunnel and a small hydrophilic region located close to FAD. In particular, compound **5** can efficiently interact with the catalytic sites of MAO A through a series of hydrophobic interactions with specific protein residues, namely Ile 180, Ile 325, and Ile 335. It is worth mentioning the formation of a π–π stacking with Phe 208 and a CH/π interaction with Pro 113 (Figure 4). These two interactions involve two aromatic rings in the center of compound **5** moiety, substantially stabilizing the interaction with MAO active site and accounting for the difference in the inhibitory potency of compound **5** in comparison to compound **2** (Ki < 1 μM for **5** vs. Ki > 200 μM for **2**). As a result, replacing the inner dipiperidine moiety of compound **2** with the dianilide structure in compound **5** is crucial for enhancing the inhibitory potential of this class of polyamine analogs.

These data confirme that the introduction of a dianiline moiety (compound **4**) in place of a dipiperidine of compound **2** determines an improvement in the MAO inhibitory potency. Furthermore, the increase in structural stiffness due to the presence of amide groups further contributes to the stabilization of compound **5** within the MAO A catalytic pocket. Interestingly, amide groups of compound **3**, even in the absence of aromatic rings, significantly increase the potency with respect to compound **2**, while in compounds **4** and **5**, both bearing inner aromatic groups, the modification of the dianiline moiety of compound **4** in a dianilide of compound **5** increased only slightly the potency, indicating in this couple of compounds the major contribution of the feature of the central core instead of the importance of the basicity of the inner nitrogen atoms.

### 2.4. Biological Evaluation

#### 2.4.1. Antiproliferative Activity

**Figure 5:**
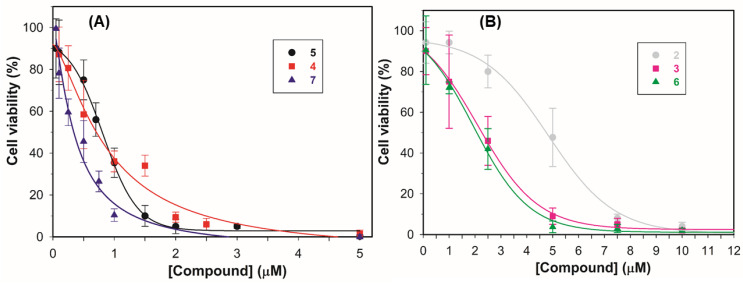
Cell viability percentage curves as a function of compound concentration (μM). LN-229 cells were incubated for 48 h in the presence of compounds **4**, **5**, and **7** (**A**) or **2**, **3**, and **6** (**B**) at different concentrations (0.1–10 μM). Data are the means ± SD of five independent experiments in duplicate. The GI_50_ values were determined by fitting the standard four-parameter logistic curve to the percentage of cell viability–compound concentration data using Sigma Plot 9.0 software. The reports of best fits were: r^2^ > 0.99 for compounds **2**–**6** and r^2^ = 0.97 for compound **4**; *p* < 0.005 was obtained in the analysis of variance corrected for the mean of the observations for all the tested compounds.

In previous studies, we found that some naphthalene diimide derivatives, including compound **7**, were able to induce cytotoxic effects [36]. Moreover, MAO A and MAO B inhibitors were found to decrease the glioma progression [17,45,46], supporting the hypothesis of a critical role played by MAOs in mediating oncogenesis in high-grade gliomas [46]. On this basis, we investigated the antiproliferative effect of compounds **1**–**7** on LN-229, a human glioblastoma cell line. Compound **1** and the well-known antitumor drug doxorubicin were taken as references. The GI_50_ values, representing the concentration of compound inducing a reduction of 50% in the cell number with respect to the control culture, are shown in Table 3. The cell viability percentage of LN-229 as a function of the concentration of **2**–**7** is shown in Figure 5. The data in Table 3 support the lack of cytotoxicity of compound **1**. Interestingly, the replacement of the inner octamethylene chain of compound **1** with the less flexible dipiperidine moiety (in compounds **2** and **3**) led to a significant increase in the antiproliferative activity regardless the nature of the inner nitrogen atoms.

Moreover, the replacement of the dipiperidine in compounds **2** and **3** with the dianiline moiety to obtain compounds **4** and **5**, respectively, caused a further increase in the cytotoxic ability as assessed by the low micromolar GI_50_ values obtained for both these latter derivatives. The enlargement of the central moiety with a tetracyclic ring system (**6** and **7**) preserved the antiproliferative effectiveness. In particular, compound **7**, characterized by a naphthalene diimide core, was the most cytotoxic of the series (GI_50_ = 0.4 ± 0.1 μM), in agreement with previous data obtained for other tumor cells [36]. The analog **6**, lacking the imide groups, appeared to be significantly less effective in inducing the cell effect even if a GI_50_ in the micromolar range was obtained.

In this connection, the presence of the amide moiety in the central core also appeared to be beneficial in inducing the cytotoxicity for the pair of compounds **2** and **3**. Otherwise, the amide did not play a relevant role in the effects induced by **4** and **5**, which showed similar GI_50_ values. Overall, among the tested compounds, **4**, **5**, and **7** were found to be the most effective as antiproliferative agents (Table 3). Further cellular investigations with other tumor cell lines and specific non-tumorigenic/healthy cell lines will be necessary for a more in-depth evaluation of the selectivity of these compounds as antiproliferative agents for potential pharmacological applications.

#### 2.4.2. Compounds **4** and **5** on MAO Activity in LN-229 Lysates

**Figure 6:**
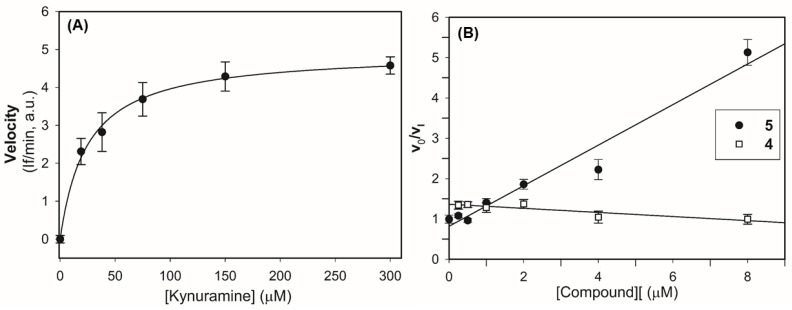
MAO activity and effects of compounds **4** and **5** in LN-229 lysates. (**A**) MAO activity at various substrate concentrations. The continuous line is the result of the best fit of the Michaelis–Menten equation to the experimental data. (**B**) Effects of compounds **4** and **5** on MAO activity in LN-229 lysates. The ratio between the velocity in the absence (Vo) and in the presence (V_I_) of test compound is plotted vs. the compound concentration. Straight lines are the results of the linear regression analysis (for fit of compound **5** data, r > 0.99).

Based on the interesting biological profile shown by compounds **4** and **5** both as inhibitors of human recombinant MAOs (Table 1) and as antiproliferative agents in LN-229 glioblastoma cells (Table 3), we investigated their inhibitory effects on the enzyme activity in cell lysates. For this purpose, we performed a preliminary analysis to verify the presence of MAO activity in LN-229 lysates using various concentrations of kynuramine as the substrate. The results reported in Figure 6A clearly show an increase in the oxidative deamination of kynuramine upon increasing the substrate concentration, with a saturating effect at high concentrations. By fitting the Michaelis–Menten equation to the experimental data, the following kinetic parameters were obtained: apparent K_M_ = 25 ± 3 μM and apparent V_max_ = 4.96 ± 2.57 If/min (in a.u.), corresponding to 20.5 pmol_(4-HQ)_ min^−1^ mg_protein_^−1^.

To evaluate the contribution of the two MAO isoforms to the total MAO activity in lysates, specific inhibitors of MAO A and MAO B were used. In detail, the lysates were pre-incubated for 15 min with clorgyline (an irreversible and specific inhibitor of MAO A), deprenyl (an irreversible and specific inhibitor of MAO B), and pargyline (an irreversible inhibitor of both MAO isoforms) [47] before determining the residual MAO activity by adding kynuramine (300 μM) as the substrate. The results shown in Table 4 clearly demonstrate that after pre-incubation with pargyline, only about 3% of the residual activity was detected, confirming that the enzymatic activity on kynuramine depended only on the MAOs. The percentage of inhibition after clorgyline treatment suggested that 75% of the MAO activity was due to the MAO A isoform and that the residual 25% was due to the MAO B isoform being inhibited by deprenyl. These data also were confirmed by treatment with safinamide, a specific and reversible MAO B inhibitor (Ki = 20 nM on human recombinant MAO B), and harmine, a reversible MAO A inhibitor (Ki = 2 nM on human recombinant MAO A).

Thus, the effects of compounds **4** and **5** on the MAO activity were also evaluated in LN-229 cell lysates using kynuramine as the substrate (at a 20 μM concentration; that is, slightly lower than the apparent K_M_ determined in lysates) and the test compounds in the range of 0.05–8 μM. As shown in Figure 6B, in these experimental conditions, derivative **5** induced a concentration-dependent inhibition with a calculated Ki = 2 μM. On the other hand, the MAO activity was not affected by increasing the concentration of compound **4** (Figure 6B). These results suggested that compound **5**, the most effective inhibitor for both the human MAO isoforms (Table 1), also was effective in lysate. As compounds **4** and **5** induced a similar cytotoxic effect (Table 2), it is reasonable to hypothesize that **4** may affect cell viability by interacting with some cellular targets/biomolecules other than MAOs.

## 3. Discussion

Monoamine oxidases, well-known pharmacological targets in neurodegenerative and neurological disorders, have recently emerged as potential targets in cancer. Indeed, MAOs (mainly the MAO A isoform) were found to be increased/overexpressed in certain types of tumors, such as prostate cancer and glioblastoma [7,8,12,19]. Therefore, MAO inhibition in relation to cancer regression is an object of investigation for the potential development of novel anticancer therapies [7,16,21,48,49,50,51].

Starting from our previous studies on the polyamine analog **2** as a reversible MAO B inhibitor, five structurally related derivatives (compounds **3**–**7**) were selected. These compounds, which were characterized by a reduced flexibility of the inner moiety compared to the lead compound, were evaluated as MAOIs with the aims to investigate how these modifications affect their inhibitory ability and to outline the structure–activity relationships. The replacement of the inner dipiperidine moiety of compound **2** with a less flexible dianiline moiety (compound **4**) and the further modification of the amine functions in two amide groups (compound **5**) strongly improved their inhibitory potency, decreasing the Ki values in the submicromolar range (Ki < 1 μM); that is, by more than two orders of magnitude with respect to compound **2** (Ki > 250 μM). With regard to the mechanism of inhibition, the most active compounds (**4** and **5**) acted as reversible and mainly competitive inhibitors, as they bound with the enzyme’s active site. The presence of a large and more rigid inner core (the naphthalene diimide-related ring in compounds **6** and **7**) reduced the accessibility of the compound to the MAO active site and decreased the inhibitory potency. Docking studies highlighted the structural determinants of the inhibitory potency of compound **5** for MAO A: the hydrophobic part of its active site tunnel efficiently interacts with **5**, and the inner aromatic rings of the compound plays a key role in the formation of π–π stacking with Phe 208. Thus, the part of the molecule that makes compound **5** promising in MAO inhibitory activity seems to be the dianiline–amide core. Several different classes of compounds have been reported as good MAOIs, and many of them are molecules characterized by the presence of rings [3]; that is, a constrained, or slight flexible system, such as in β-carboline derivatives [52] and in coumarine and flavonoid derivatives, to cite only some of them. As the “rings” of these inhibitors are not inserted in a polyamine skeleton, their structures are quite different from those of analogs **4** and **5**, which can be considered as novel scaffolds.

It is worth noting that even if the improved inhibitory potency of compounds **4** and **5** (in comparison with the lead **2**) did not correspond to an improved selectivity between the two MAO isoforms (selectivity index: KiMAO A:KiMAO B = 3:1), compound **5** was found to be very specific for MAO in comparison to the two other types of AOs and the pharmacological targets SMOX and VAP-1.

Compounds **4** and **5**, the most effective MAOIs, also were endowed with notable antiproliferative effects on the LN-229 glioblastoma cells (GI_50_ < 1 μM). Taking into account the overexpression of MAOs in glioblastoma and the effects of MAOIs in glioma progression [17,45,46], the effectiveness of compounds **4** and **5** as MAO inhibitors was evaluated in LN-229 cell lysates. Surprisingly, only compound **5** also was found to be very effective in inhibiting MAO activity in lysates. This different behavior suggests that cellular targets other than MAOs can be responsible for the cytotoxicity exerted by compound **4**.

In conclusion, the main results of this study were the identification of compounds **4** and **5** as potent and reversible MAO inhibitors, and effective antiproliferative agents against the LN-229 glioblastoma cell line. As an overexpression of MAO was found in glioblastoma, the polyamine scaffold of compounds **4** and **5** is worthy of consideration for future development of multi-targeting anticancer agents.

Further studies are in progress to evaluate the tumor specificity of these analogs, to clarify the intracellular mechanism of action determining cell death in LN-229, and the role played by MAO activity in this process.

## 4. Materials and Methods

All reagents were of analytical grade and were purchased from Merk s.r.l. (Milan, Italy) with the exception of the Amplex Red reagent (10-acetyl-3,7-dihydroxyphenoxazine), which was purchased from Invitrogen (Invitrogen s.r.l, San Giuliano Milanese (Milan, Italy). The human recombinant MAO A and MAO B were expressed in baculovirus-infected BT1 cells (5 mg/mL), and horseradish peroxidases were purchased from Merk s.r.l. (Italy). The VAP-1 was a kind gift from Biothie Therapies Cor. (Turku, Finland). The recombinant SMOX protein was expressed in *E. coli* BL21 DE3 cells and purified as previously reported [40]. The stock solution of compounds **1**–**2** were prepared in milliQ water, and the other compounds were dissolved in dimethyl sulfoxide (10 mM stock concentration).

### 4.1. Chemistry

**Scheme 1:**
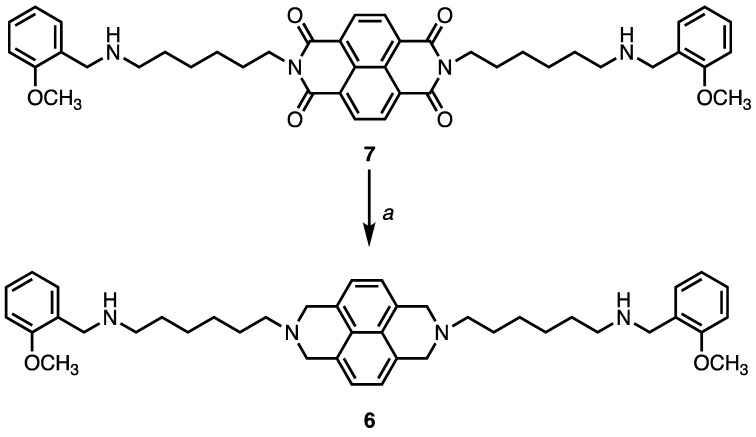
(a) A 2 M borane N-ethyl-N-isopropylaniline complex in THF, dry diglyme, reflux, 5 h, N_2_, 32% yield.

Compound **6** was synthesized following the procedure reported in Scheme 1. Briefly, a solution of 2 M of borane N-ethyl-N-isopropylaniline complex in THF (4 mL) was added to compound **7** (1.41 g, 2 mmol) in dry diglyme (20 mL) under a stream of nitrogen, and the resulting mixture was refluxed for 5 h. After cooling down, water (4 mL) and 6N HCl_aq_ (4 mL) were carefully added dropwise, and the mixture was then refluxed for 1 h. The organic solvent was removed in vacuo, and the aqueous layer was extracted with dichloromethane (3 × 20 mL), which was dried and evaporated to give a residue that was purified via flash chromatography using as the mobile phase a mixture of dichloromethane/methanol/aqueous ammonia 33% (9:1:0.05) to give 6 (0.416 g, 32% yield) as a yellow oil. ^1^H NMR (free base, 400 MHz, CDCl_3_) δ 1.13–1.27 (m, 12H), 2.69–2.83 (m, 8H), 3.88 (s, 4H), 3.97 (s, 6H), 4.12–4.32 (m, 12H), 6.84–6.99 (m, 4H), 7.30–7.37 (m, 4H), 8.78 (s, 4H); MS (ESI+) *m*/*z* = 650 (M + H)^+^. Compounds **1**–**5** and **7** were synthetized as previously reported [34,36].

### 4.2. Amine Oxidase Assay Methods

The AO activity was performed using two different assay methods. When possible, for the AO activity of VAP-1 and SMOX, a fluorometric assay that detected the H_2_O_2_ generation rate via a peroxidase-coupled continuous assay was applied. This fluorometric method used the Amplex Red reagent as the substrate for horseradish peroxidase [53]. Each assay was performed at 37 °C in a solution (800 μL) containing 0.1 M potassium phosphate buffer (pH 7.4) and 0.1 mM EDTA in the presence of Amplex Red (100 μM) and horseradish peroxidase type II (5 UmL^−1^). The initial velocities were determined by measuring the increase in fluorescence intensity (λ_exc_ = 563 nm and λ_em_ = 586 nm), and the H_2_O_2_ generation rate was calculated from the change in fluorescence intensity by means of calibration curves obtained by serial dilution of the stock solution of H_2_O_2_. Except for compound **4**, no significant interference of the other polyamine analogs with the preliminary calibration curves was observed. Benzylamine and spermine were used as the substrates for VAP-1 and SMOX, respectively. The evaluations of the effects of compounds **1**–**7** on the MAO activity were performed by using a kynuramine assay [54] due to the interferences of compound **4** with the Amplex Red method. MAO stock solutions or cell lysates were diluted with assay buffer (final concentration of 0.006 mg/mL for MAO A, 0.004 mg/mL for MAO B, or 0.3 mg/mL for LN-229 lysates, for a total volume of 0.2 mL) in the presence or absence of the various inhibitors. Then, the substrate (kynuramine) was added at a specific final concentration (10 μM for the screening with all the compounds or in the range of 10–320 μM concentrations in the experiments performed to determinate the K_M_ and V_max_). After incubation for 45 min at 37 °C, the reaction was stopped by addition of 2 M NaOH (80 μL) and 480 μL of distilled water. Kynuramine deaminated by MAOs spontaneously cyclized to give 4-hydroxyquinoline, the amount of which was determined via the fluorescence intensity of the peak of its emission spectra (λ_exc_ = 330 nm and λ_em_ =330–530 nm) using a specific calibration curve built with the standard 4-hydroxyquinoline. To determine the MAO isoform in cell lysates, samples were pre-incubated for 15 min at 37 °C in the absence or in the presence of the irreversible and specific inhibitors (5 nM clorgyline to inhibit MAO A, 5 nM deprenyl to inhibit MAO B, and 0.5 mM pargyline to inhibit both MAO isoforms) before adding the substrate at a 300 μM concentration (saturating conditions). For the assay with reversible inhibitors, MAO activity was performed in the presence of safinamide (0.2 μM to inhibit MAO B) or harmine (0.2 μM to inhibit MAO A) and 20 μM kynuramine as a non-saturating substrate.

A control sample (in the absence of the inhibitor) and a blank sample (in the absence of the enzyme) were also run under the same experimental conditions. All kinetic experiments were performed at least in triplicate.

A Cary-Eclipse fluorimeter and a Cary Scan UV-Vis spectrophotometer (Varian Inc., Palo Alto, CA, USA) were used for the fluorometric and spectrophotometric measurements, respectively.

#### Kinetic Analysis

Steady-state kinetic parameters (V_max_ and K_M_) were calculated by fitting the Michaelis–Menten equation to the experimental data (initial rate of reactions vs. substrate concentrations) with Sigma Plot software version 9.0 (Jandel Scientific, San Rafael, CA, USA). The apparent V_max_ and K_M_ values of the human recombinant MAO A and MAO B were determined in the presence of different concentrations of the various compounds. The mode of inhibition was determined with a global fit analysis (GraphPad 9.0 software, GraphPad Software, San Diego, CA, USA) of the initial rate of reaction (V_0_) vs. the substrate concentration plots in the presence and absence of the inhibitor to fit equations for competitive, mixed, non-competitive, and uncompetitive inhibition models; the fit giving the highest r^2^ value was selected for the calculation of the inhibition constants (Ki).

The reversibility of the inhibition was evaluated by incubating the enzyme (recombinant MAO A or MAO B) with **4** or **5** at a 5 μM concentration (more than fivefold with respect to their Ki values) in potassium phosphate buffer (0.1 M K/Pi, pH 7.4, and 0.1 mM EDTA) at 37 °C. After 25 min, the enzyme–inhibitor (or the control sample without the inhibitor) solution was diluted fiftyfold, and 300 μM of kynuramine was added as the substrate to measure the residual enzyme activity.

Ki values are expressed as mean ± S.D.

Linear regression analysis was performed by using Sigma Plot version 9.0 (Jandel Scientific, San Rafael, CA, USA); unless stated otherwise, the correlation coefficient for the linear regression was 0.98 or greater.

### 4.3. Cell Culture

The LN-229 (human glioblastoma) was purchased from the American Type Culture Collection (ATCC) and cultured in DMEM (D2902, Sigma Chemical Co., St. Louis, MO, USA) supplemented with 3.5 g/L of glucose and 5% heat-inactivated fetal calf serum (FCS) (F7524, Sigma Chemical Co.), respectively. A total of 100 U/mL of penicillin, 100 μg/mL of streptomycin, and 0.25 μg/mL of amphotericin B (Sigma Chemical Co.) were added to the medium. Cells were cultured at 37 °C in a humidified atmosphere incubator containing 5% carbon dioxide in air.

### 4.4. Cell Lysate

The LN-229 (about 9 × 10^5^) was seeded in standard conditions. After 24 h (80% confluence), cells were collected, trypsinized, and washed twice with phosphate buffer saline (PBS)-EDTA (10 mM Na_2_HPO_4_, 1.8 mM KH_2_PO_4_, 2.7 mM KCl, 137 mM NaCl, and 1 mM EDTA) at 4 °C and frozen in liquid nitrogen until use. About 10 million cells were lysed in 1 mL of lysis buffer (20 mM Hepes (pH 7.4), 1 mM EDTA, and protease inhibitor cocktail (1:400 *v*/*v*)) and frozen in liquid nitrogen until use. The protein content was measured by using the Bradford method with bovine serum albumin as the standard [55].

### 4.5. Inhibition Growth Assay

Cells (3–6 × 10^4^/well) were seeded into a 24-well cell culture plate; after 24 h, different concentrations of the test compounds or reference drug were added. After 48 h of incubation in standard conditions, the cell viability was determined by using a Trypan blue exclusion assay. Briefly, cells were detached by using a 10 mM trypsin and 0.3 mM EDTA solution in PBS and stained with 0.1% Trypan blue solution in PBS. Unstained viable cells were immediately counted in a Burker chamber. The percentage of viable cells was calculated with respect to the untreated control culture. Antiproliferative data were expressed as GI_50_ values; that is, the concentration of the test agent that induced a 50% reduction in the cell number compared to a control untreated culture. The GI_50_ concentrations were calculated via non-linear regression analysis by fitting the standard four-parameter logistic curve to the data using Sigma Plot version 9.0 (Jandel Scientific, San Rafael, CA, USA). The goodness of the fit was expressed by the r^2^ coefficient, and the best fit report included the analysis of variance corrected for the mean of the observations with the *p* values (*p* < 0.005 for all the tested compounds). Data are the means ± SD of at least five independent experiments in duplicate.

### 4.6. In Silico Analysis

The crystal structure of the human MAO A was retrieved from the Protein Data Bank (PDB code: 2Z5X) and processed in order to remove ligands and unnecessary water molecules. Hydrogen atoms were added using standard geometries with the MOE program [56]. To minimize contacts between hydrogens, the structures were subjected to FF19SB force field minimization until the root-mean-square deviation of the conjugate gradient was <0.1 kcal•mol^−1^•Å^−1^ (1 Å = 0.1 nm), keeping the heavy atoms fixed at their crystallographic positions. Selected compounds were built as minimized and charged using the MMFF94x force field of MOE. Before the molecular docking procedure, a Site Finder approach [56] was performed. Using the Site Finder indications, docking experiments were performed using the MOE Dock program. Triangle Matcher was exploited as the placement method, while GBVI/WSA dG was used as the scoring tool. The docking complexes were subjected to a molecular dynamics (MD) simulation using ACEMD [57] (FF19SB force field) with explicit water molecules (TIP3P model). Firstly, a solvent equilibration (1 ns) was obtained by applying positional restraints on carbon atoms. Secondly, 10 ns molecular dynamics simulations were performed on the full system. The equilibration phase was performed by using NPT ensemble (isothermal and isobaric) at a constant pressure (1 atm; Berendsen method) and temperature (300 K; Langevin thermostat).

## Figures and Tables

**Table 1:** Inhibition constant values and selectivity index (SI) of polyamine analogs **1**–**7** toward human recombinant MAO A and MAO B. Safinamide, harmine, and isatine were taken as reference competitive inhibitors.

Compound	Structure	MAO A Ki (μM)	MAO B Ki (μM)	SI ^a^
**1**	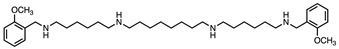	290 ± 60	470 ± 50	1:1.6
**2**	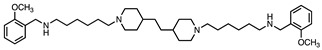	247 ± 42	330 ± 30	1:1.3
**3**	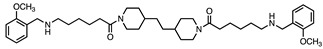	70 ± 7	19 ± 2	3.7:1
**4**	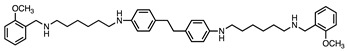	0.9 ± 0.2	0.3 ± 0.1	3:1
**5**	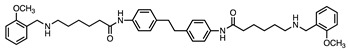	0.6 ± 0.1	0.2 ± 0.1	3:1
**6**	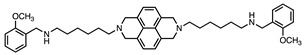	37 ± 4	27 ± 4	1.6:1
**7**	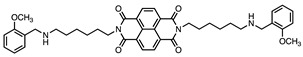	3.2 ± 0.2	4.2 ± 0.3	1:1.3
Safinamide	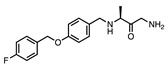	>10	(23 ± 4) × 10^−3^	-
Isatine	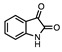	16 ± 3	4 ± 1	4:1
Harmine	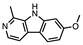	(2 ± 4) × 10^−3^	>10	-

^a^ Selectivity index: Ki_MAO A_:Ki_MAO B._

**Table 2:** Effects of **1**–**3** and **5**–**7** on the catalytic efficiency (V_max_/K_M_) of human VAP-1 and murine SMOX in comparison to those on MAOs.

	Vmax/K_M_ Relative to Control			
	VAP-1	SMOX	MAO A	MAO B
Compound ^1^	50 μM ^1^	10 μM	10 μM	10 μM
**1**	1	0.21	1.0	1.0
**2**	0.60	0.61	1.0	1.0
**3**	0.37	0.47	0.95	0.64
**5**	0.48	0.46	0.06	0.02
**6**	0.50	0.21	0.22	0.30
**7**	0.55	0.16	0.71	0.75

^1^ For VAP-1, compounds were tested at 50 μM, as no inhibition was observed at a 10 μM concentration.

**Table 3:** Antiproliferative effects of compounds **2**–**7** on the LN-229 glioblastoma cell line after 48 h of incubation. Compound **1** and the antitumor drug doxorubicin (DOXO) were taken as references.

	LN-229
Compounds	GI_50_ (μM) ^1^
**1**	>20
**2**	4.6 ± 1.1
**3**	2.2 ± 0.9
**4**	0.9 ± 0.2
**5**	0.8 ± 0.1
**6**	2.0 ± 0.9
**7**	0.4 ± 0.1
DOXO	0.012 ± 0.004

^1^ GI_50_ values are the mean ± SD of five independent experiments in duplicate.

**Table 4:** Effects of standard inhibitors on MAO activity in LN-229 cell lysates.

Inhibitor	Residual MAO Activity in Lysates
Deprenyl ^1^ (5 nM)	0.74 ± 0.04
Clorgyline ^1^ (5 nM)	0.34 ± 0.10
Pargyline ^1^ (0.5 mM)	0.03 ± 0.02
Safinamide ^1^ (0.2 μM)	0.66 ± 0.04
Harmine ^1^ (0.2 μM)	0.37 ± 0.08

^1^ Monoamine oxidase specific activity in control sample (100%): 20.5 pmol_(4-HQ)_ min^−1^ mg_protein_^−1^, tested with 300 μM of kynuramine as the substrate for evaluating the effect of deprenyl, clorgyline and pargyline as inhibitors; monoamine oxidase specific activity: 5 pmol_(4-HQ)_ min^−1^ mg_protein_^−1^, tested with 20 μM of kynuramine, for evaluating safinamide and harmine as inhibitors.

## Data Availability

The data are available from the corresponding authors upon reasonable request.

## Supplementary Materials

The following supporting information can be downloaded at: https://www.mdpi.com/article/10.3390/molecules28176329/s1, Figure S1: Double reciprocal plots of MAO B activity in the presence of various concentrations of compound **7**.

## References

[1] Tipton K.F. (2018). 90 years of monoamine oxidase: Some progress and some confusion. J. Neural. Transm..

[2] Youdim M.B., Edmondson D., Tipton K.F. (2006). The therapeutic potential of monoamine oxidase inhibitors. Nat. Rev. Neurosci..

[3] Tripathi A.C., Upadhyay S., Paliwal S., Saraf S.K. (2018). Privileged scaffolds as MAO inhibitors: Retrospect and prospects. Eur. J. Med. Chem..

[4] Carradori S., Fantacuzzi M., Ammazzalorso A., Angeli A., De Filippis B., Galati S., Petzer A., Petzer J.P., Poli G., Tuccinardi T. (2022). Resveratrol Analogues as Dual Inhibitors of Monoamine Oxidase B and Carbonic Anhydrase VII: A New Multi-Target Combination for Neurodegenerative Diseases?. Molecules.

[5] Santin Y., Resta J., Parini A., Mialet-Perez J. (2021). Monoamine oxidases in age-associated diseases: New perspectives for old enzymes. Ageing Res. Rev..

[6] Chen C.H., Wu B.J. (2023). Monoamine oxidase A: An emerging therapeutic target in prostate cancer. Front. Oncol..

[7] Meenu M., Verma V.K., Seth A., Sahoo R.K., Gupta P., Arya D.S. (2020). Association of Monoamine Oxidase A with Tumor Burden and Castration Resistance in Prostate Cancer. Curr. Ther. Res. Clin. Exp..

[8] Shih J.C. (2018). Monoamine oxidase isoenzymes: Genes, functions and targets for behavior and cancer therapy. J. Neural. Transm..

[9] Wang Y.C., Wang X., Yu J., Ma F., Li Z., Zhou Y., Zeng S., Ma X., Li Y.R., Neal A. (2021). Targeting monoamine oxidase A-regulated tumor-associated macrophage polarization for cancer immunotherapy. Nat. Commun..

[10] Liu F., Hu L., Ma Y., Huang B., Xiu Z., Zhang P., Zhou K., Tang X. (2018). Increased expression of monoamine oxidase A is associated with epithelial to mesenchymal transition and clinicopathological features in non-small cell lung cancer. Oncol. Lett..

[11] Flamand V., Zhao H., Peehl D.M. (2010). Targeting monoamine oxidase A in advanced prostate cancer. J. Cancer Res. Clin. Oncol..

[12] Gabilondo A.M., Hostalot C., Garibi J.M., Meana J.J., Callado L.F. (2008). Monoamine oxidase B activity is increased in human gliomas. Neurochem. Int..

[13] Dhabal S., Das P., Biswas P., Kumari P., Yakubenko V.P., Kundu S., Cathcart M.K., Kundu M., Biswas K., Bhattacharjee A. (2018). Regulation of monoamine oxidase A (MAO-A) expression, activity, and function in IL-13-stimulated monocytes and A549 lung carcinoma cells. J. Biol. Chem..

[14] Wu J.B., Shao C., Li X., Li Q., Hu P., Shi C., Li Y., Chen Y.T., Yin F., Liao C.P. (2014). Monoamine oxidase A mediates prostate tumorigenesis and cancer metastasis. J. Clin. Investig..

[15] Peehl D.M., Coram M., Khine H., Reese S., Nolley R., Zhao H. (2008). The significance of monoamine oxidase-A expression in high grade prostate cancer. J. Urol..

[16] Gross M.E., Agus D.B., Dorff T.B., Pinski J.K., Quinn D.I., Castellanos O., Gilmore P., Shih J.C. (2021). Phase 2 trial of monoamine oxidase inhibitor phenelzine in biochemical recurrent prostate cancer. Prostate Cancer Prostatic Dis..

[17] Kushal S., Wang W., Vaikari V.P., Kota R., Chen K., Yeh T.S., Jhaveri N., Groshen S.L., Olenyuk B.Z., Chen T.C. (2016). Monoamine oxidase A (MAO A) inhibitors decrease glioma progression. Oncotarget.

[18] Huang B., Zhou Z., Liu J., Wu X., Li X., He Q., Zhang P., Tang X. (2020). The role of monoamine oxidase A in HPV-16 E7-induced epithelial-mesenchymal transition and HIF-1α protein accumulation in non-small cell lung cancer cells. Int. J. Biol. Sci..

[19] Sharpe M.A., Baskin D.S. (2016). Monoamine oxidase B levels are highly expressed in human gliomas and are correlated with the expression of HiF-1α and with transcription factors Sp1 and Sp3. Oncotarget.

[20] Yang Y.C., Chien M.H., Lai T.C., Su C.Y., Jan Y.H., Hsiao M., Chen C.L. (2020). Monoamine Oxidase B Expression Correlates with a Poor Prognosis in Colorectal Cancer Patients and Is Significantly Associated with Epithelial-to-Mesenchymal Transition-Related Gene Signatures. Int. J. Mol. Sci..

[21] Aljanabi R., Alsous L., Sabbah D.A., Gul H.I., Gul M., Bardaweel S.K. (2021). Monoamine Oxidase (MAO) as a Potential Target for Anticancer Drug Design and Development. Molecules.

[22] Wang X., Li B., Kim Y.J., Wang Y.C., Li Z., Yu J., Zeng S., Ma X., Choi I.Y., Di Biase S. (2021). Targeting monoamine oxidase A for T cell-based cancer immunotherapy. Sci. Immunol..

[23] Mehndiratta S., Qian B., Chuang J.Y., Liou J.P., Shih J.C. (2022). N-Methylpropargylamine-Conjugated Hydroxamic Acids as Dual Inhibitors of Monoamine Oxidase A and Histone Deacetylase for Glioma Treatment. J. Med. Chem..

[24] Lee H.T., Choi M.R., Doh M.S., Jung K.H., Chai Y.G. (2013). Effects of the monoamine oxidase inhibitors pargyline and tranylcypromine on cellular proliferation in human prostate cancer cells. Oncol. Rep..

[25] Minarini A., Milelli A., Tumiatti V., Rosini M., Bolognesi M.L., Melchiorre C. (2010). Synthetic polyamines: An overview of their multiple biological activities. Amino Acids.

[26] Karigiannis G., Papaioannou D. (2000). Structure, biological activity and synthesis of polyamine analogues and conjugates. Eur. J. Org. Chem..

[27] Casero R., Marton L. (2007). Targeting polyamine metabolism and function in cancer and other hyperproliferative diseases. Nat. Rev. Drug. Discov..

[28] Casero R.A., Woster P.M. (2009). Recent advances in the development of polyamine analogues as antitumor agents. J. Med. Chem..

[29] Casero R.A., Murray Stewart T., Pegg A.E. (2018). Polyamine metabolism and cancer: Treatments, challenges and opportunities. Nat. Rev. Cancer.

[30] Dobrovolskaite A., Gardner R.A., Delcros J.G., Phanstiel O. (2022). Development of Polyamine Lassos as Polyamine Transport Inhibitors. ACS Med. Chem. Lett..

[31] Wang J., Kaiser M., Copp B.R. (2014). Investigation of Indolglyoxamide and Indolacetamide Analogues of Polyamines as Antimalarial and Antitrypanosomal Agents. Mar. Drugs.

[32] Sharma S.K., Hazeldine S., Crowley M.L., Hanson A., Beattie R., Varghese S., Senanayake T.M.D., Hirata A., Hirata F., Huang Y. (2012). Polyamine-based small molecule epigenetic modulators. Med. Chem. Commun..

[33] Houdou M., Jacobs N., Coene J., Azfar M., Vanhoutte R., Van den Haute C., Eggermont J., Daniëls V., Verhelst S.H.L., Vangheluwe P. (2023). Novel Green Fluorescent Polyamines to Analyze ATP13A2 and ATP13A3 Activity in the Mammalian Polyamine Transport System. Biomolecules.

[34] Tumiatti V., Minarini A., Milelli A., Rosini M., Buccioni M., Marucci G., Ghelardini C., Bellucci C., Melchiorre C. (2007). Structure-activity relationships of methoctramine-related polyamines as muscarinic antagonist: Effect of replacing the inner polymethylene chain with cyclic moieties. Bioorg. Med. Chem..

[35] Bonaiuto E., Minarini A., Tumiatti V., Milelli A., Lunelli M., Pegoraro M., Rizzoli V., Di Paolo M.L. (2012). Synthetic polyamines as potential amine oxidase inhibitors: A preliminary study. Amino Acids.

[36] Tumiatti V., Milelli A., Minarini A., Micco M., Gasperi Campani A., Roncuzzi L., Baiocchi D., Marinello J., Capranico G., Zini M. (2009). Design, synthesis, and biological evaluation of substituted naphthalene imides and diimides as anticancer agent. J. Med. Chem..

[37] Binda C., Wang J., Pisani L., Caccia C., Carotti A., Salvati P., Edmondson D.E., Mattevi A. (2007). Structures of human monoamine oxidase B complexes with selective noncovalent inhibitors: Safinamide and coumarin analogs. J. Med. Chem..

[38] Hubálek F., Binda C., Khalil A., Li M., Mattevi A., Castagnoli N., Edmondson D.E. (2005). Demonstration of isoleucine 199 as a structural determinant for the selective inhibition of human monoamine oxidase B by specific reversible inhibitors. J. Biol. Chem..

[39] Kim H., Sablin S.O., Ramsay R.R. (1997). Inhibition of monoamine oxidase A by beta-carboline derivatives. Arch. Biochem. Biophys..

[40] Di Paolo M.L., Cervelli M., Mariottini P., Leonetti A., Polticelli F., Rosini M., Milelli A., Basagni F., Venerando R., Agostinelli E. (2019). Exploring the activity of polyamine analogues on polyamine and spermine oxidase: Methoctramine, a potent and selective inhibitor of polyamine oxidase. J. Enzyme Inhib. Med. Chem..

[41] Pannecoeck R., Serruys D., Benmeridja L., Delanghe J.R., van Geel N., Speeckaert R., Speeckaert M.M. (2015). Vascular adhesion protein-1: Role in human pathology and application as a biomarker. Crit. Rev. Clin. Lab. Sci..

[42] Salmi M., Jalkanen S. (2019). Vascular Adhesion Protein-1: A Cell Surface Amine Oxidase in Translation. Antioxid. Redox Signal..

[43] Hu T., Sun D., Zhang J., Xue R., Janssen H.L.A., Tang W., Dong L. (2018). Spermine oxidase is upregulated and promotes tumor growth in hepatocellular carcinoma. Hepatol. Res..

[44] Kim S., Kim D., Roh S., Hong I., Kim H., Ahn T.S., Kang D.H., Lee M.S., Baek M.-J., Kwak H.J. (2022). Expression of Spermine Oxidase Is Associated with Colorectal Carcinogenesis and Prognosis of Patients. Biomedicines.

[45] Sjöberg R.L., Wu W.Y., Dahlin A.M., Tsavachidis S., Bondy M.L., Melin B., Gliogene Group (2019). Role of monoamine-oxidase-A-gene variation in the development of glioblastoma in males: A case control study. J. Neurooncol..

[46] Marconi G.D., Gallorini M., Carradori S., Guglielmi P., Cataldi A., Zara S. (2019). The Up-Regulation of Oxidative Stress as a Potential Mechanism of Novel MAO-B Inhibitors for Glioblastoma Treatment. Molecules.

[47] Fowler C.J., Mantle T.-J., Tipton K.F. (1982). The nature of the inhibition of rat liver monoamine oxidase types A and B by the acetylenic inhibitors clorgyline, l-deprenyl and pargyline. Biochem. Pharmacol..

[48] Zarmouh N.O., Messeha S.S., Mateeva N., Gangapuram M., Flowers K., Eyunni S.V.K., Zhang W., Redda K.K., Soliman K.F.A. (2020). The Antiproliferative Effects of Flavonoid MAO Inhibitors on Prostate Cancer Cells. Molecules.

[49] Resta J., Santin Y., Roumiguié M., Riant E., Lucas A., Couderc B., Binda C., Lluel P., Parini A., Mialet-Perez J. (2022). Monoamine Oxidase Inhibitors Prevent Glucose-Dependent Energy Production, Proliferation and Migration of Bladder Carcinoma Cells. Int. J. Mol. Sci..

[50] Wan K., Luo J., Yeh S., You B., Meng J., Chang P., Niu Y., Li G., Lu C., Zhu Y. (2020). The MAO inhibitors phenelzine and clorgyline revert enzalutamide resistance in castration resistant prostate cancer. Nat. Commun..

[51] Jacobs M., Olivero J., Choi H.O., Liao C.P., Kashemirov B.A., Katz J., Gross M.E., McKenna C.E. (2023). Synthesis and anti-cancer potential of potent peripheral MAOA inhibitors designed to limit blood: Brain penetration. Bioorg. Med. Chem..

[52] Benny F., Kumar S., Jayan J., Abdelgawad M.A., Ghoneim M.M., Kumar A., Manoharan A., Susan R., Sudevan S.T., Mathew B. (2023). Review of β-carboline and its derivatives as selective MAO-A inhibitors. Arch. Pharm..

[53] Zhou M., Panchuk-Voloshina N. (1997). A one-step fluorometric method for the continuous measurement of monoamine oxidase activity. Anal. Biochem..

[54] Santillo M.F., Liu Y., Ferguson M., Vohra S.N., Wiesenfeld P.L. (2014). Inhibition of monoamine oxidase (MAO) by beta-carbolines and their interactions in live neuronal (PC12) and liver (HuH-7 and MH1C1) cells. Toxicol. In Vitro.

[55] Bradford M.M. (1976). A rapid and sensitive method for the quantitation of microgram quantities of protein utilizing the principle of protein-dye binding. Anal. Biochem..

[56] (2020). Molecular Operating Environment (MOE), 2020.09 Chemical Computing Group ULC, 1010 Sherbooke St. West, Suite #910, Montreal, QC, Canada, H3A 2R7. https://www.chemcomp.com/Products.htm.

[57] Harvey M.J., Giupponi G.V., Fabritiis G.D. (2009). ACEMD: Accelerating biomolecular dynamics in the microsecond time scale. J. Chem. Theor. Comput..

