# Supplementary material for: From Monoamine Oxidase Inhibition to Antiproliferative Activity: New Biological Perspectives for Polyamine Analogs
Source: Molecules. 2023 Aug 29;28(17):6329. doi: 10.3390/molecules28176329 (PMC10490032; doi:10.3390/molecules28176329)
Supplement: Supplementary file 1 [file molecules-28-06329-s001.zip › molecules-2562834-supplementary.pdf]

Supplementary Materials

From Monoamine Oxidase Inhibition to Antiproliferative Activity: New Biological Perspectives for Polyamine Analogs

Giulia Nordio [1,2], Francesco Piazzola [1], Giorgio Cozza [3], Monica Rossetto [3], Manuela Cervelli [4], Anna Minarini [5], Filippo Basagni [5], Elisa Tassinari [6], Lisa Dalla Via [1,2,], Andrea Milelli [6,*] and Maria Luisa Di Paolo [3,*]

[1] Department of Pharmaceutical and Pharmacological Sciences, University of Padova, 35131 Padova, Italy; giulia.nordio.1@phd.unipd.it (G.N.); francesco.piazzola@studenti.unipd.it (F.P.)
[2] Consorzio Interuniversitario Nazionale per la Scienza e Tecnologia dei Materiali (INSTM), 50121 Firenze, Italy
[3] Department of Molecular Medicine, University of Padova, 35131 Padova, Italy; giorgio.cozza@unipd.it (G.C.); monica.rossetto@unipd.it (M.R.)
[4] Department of Science, University of Rome "Roma Tre", 00146 Rome, Italy; manuela.cervelli@uniroma3.it
[5] Department of Pharmacy and Biotechnology, Alma Mater Studiorum-University of Bologna, 40126 Bologna, Italy; anna.minarini@unibo.it (A.M.); filippo.basagni2@unibo.it (F.B.)
[6] Department for Life Quality Studies, Alma Mater Studiorum-University of Bologna, 47921 Rimini, Italy; elisa.tassinari9@unibo.it
* Correspondence: andrea.milelli3@unibo.it (A.M.); marialuisa.dipaolo@unipd.it (M.L.D.P.)

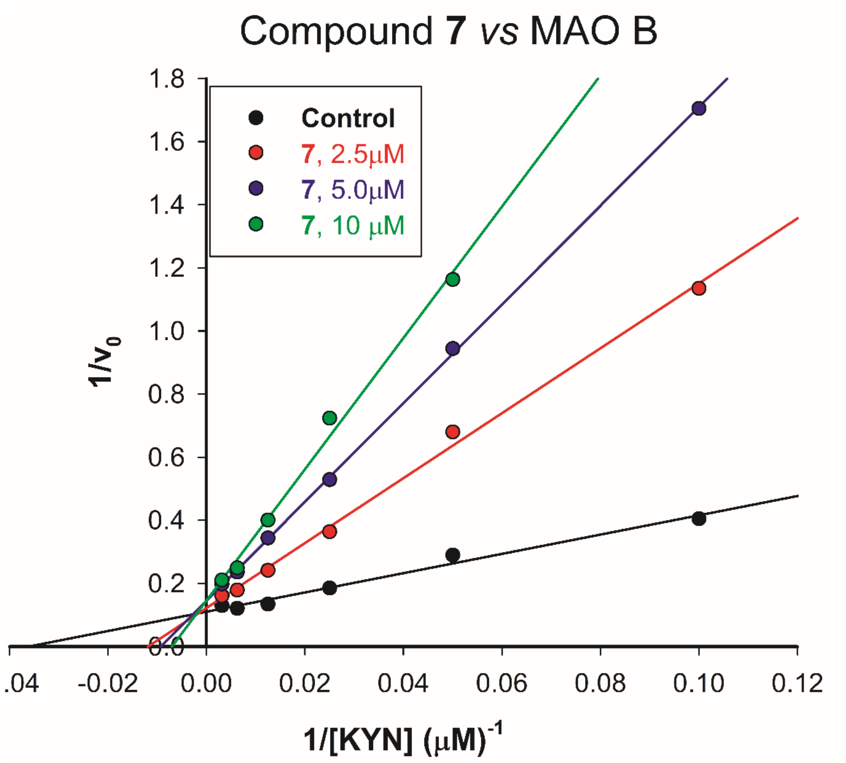

Figure S1 Double reciprocal plots of MAO B activity in the presence of various concentrations of compound **7.** Continuous lines are the results of the linear regression analysis of the plotted data (r>0.99)
